# The Grad Cohort Workshop: Evaluating an Intervention to Retain Women Graduate Students in Computing

**DOI:** 10.3389/fpsyg.2016.02071

**Published:** 2017-01-10

**Authors:** Jane G. Stout, Burçin Tamer, Heather M. Wright, Lori A. Clarke, Sandhya Dwarkadas, Ayanna M. Howard

**Affiliations:** ^1^Computing Research AssociationWashington, DC, USA; ^2^College of Information and Computer Sciences, University of Massachusetts AmherstAmherst, MA, USA; ^3^Department of Computer Science, University of RochesterRochester, NY, USA; ^4^School of Electrical and Computer Engineering, Georgia Institute of TechnologyAtlanta, GA, USA

**Keywords:** gender, computing, graduate students, role models, intervention

## Abstract

Women engaged in computing career tracks are vastly outnumbered by men and often must contend with negative stereotypes about their innate technical aptitude. Research suggests women's marginalized presence in computing may result in women psychologically disengaging, and ultimately dropping out, perpetuating women's underrepresentation in computing. To combat this vicious cycle, the Computing Research Association's Committee on the Status of Women in Computing Research (CRA-W) runs a multi-day mentorship workshop for women graduate students called Grad Cohort, which consists of a speaker series and networking opportunities. We studied the long-term impact of Grad Cohort on women Ph.D. students' (a) dedication to becoming well-known in one's field, and giving back to the community (*professional goals*), (b) the degree to which one feels computing is an important element of “who they are” (*computing identity)*, and (c) beliefs that computing skills are innate (*entity beliefs*). Of note, entity beliefs are known to be demoralizing and can lead to disengagement from academic endeavors. We compared a propensity score matched sample of women and men Ph.D. students in computing programs who had never participated in Grad Cohort to a sample of past Grad Cohort participants. Grad Cohort participants reported interest in becoming well-known in their field to a greater degree than women non-participants, and to an equivalent degree as men. Also, Grad Cohort participants reported stronger interest in giving back to the community than their peers. Further, whereas women non-participants identified with computing to a lesser degree than men and held stronger entity beliefs than men, Grad Cohort participants' computing identity and entity beliefs were equivalent to men. Importantly, stronger entity beliefs predicted a weaker computing identity among students, with the exception of Grad Cohort participants. This latter finding suggests Grad Cohort may shield students' computing identity from the damaging nature of entity beliefs. Together, these findings suggest Grad Cohort may fortify women's commitment to pursuing computing research careers and move the needle toward greater gender diversity in computing.

“I actually feel like I'm part of the computer science field now [that I have attended Grad Cohort]. I don't feel so isolated or insignificant as a female in the field…” *– Grad Cohort Participant, 2014*

“[One of my favorite things about Grad Cohort was] meeting so many other women who liked similar things as me! For the first time, I didn't feel like it was weird to be a woman in computer science.” *– Grad Cohort Participant, 2016*

The quotes by the graduate students above illustrate the field of computing can be alienating for women. Indeed, social science research indicates women who have opted into a computing career path must regularly contend with negative stereotypes about their technical abilities (e.g., Miller et al., 2015), as well as the belief that women do not “fit” in computing to the degree that men do (Cheryan et al., 2013). The cultural stereotypes that computer science is for men is reinforced by the fact that most students, post-docs, college professors, and other professionals in the field are men (National Science Foundation, 2015a,b, 2016a,b,c,d), as are portrayals of these professionals in the media (Smith et al., 2014).

That women are vastly underrepresented at all levels of the computing education and career pipeline is problematic for a number of reasons. First, diversity in the workplace is known to foster innovation; a diversity of experiences and perspectives yields greater opportunity for creativity (Leung et al., 2008; Woolley et al., 2010; Hoever et al., 2012). Second, a homogenous set of prerogatives at the decision table leaves unrepresented voices unheard, so that the needs of many are ignored. For example, early voice activated systems developed by computer scientists only worked for men because women's voices were literally unheard during development (Margolis and Fisher, 2002; Camp, 2012). Finally, computing professions tend to be financially lucrative and culturally valued (Forbes, 2016; U.S. News World Report, 2016). Thus, low representation of women in computing may perpetuate social inequality among women and men (Cejka and Eagly, 1999; Sheffield, 2004). In sum, a dearth of women in computing careers poses a host of problems for our culture.

The Computing Research Association's Committee on the Status of Women in Computing Research (CRA-W) designs and runs programs to boost the number of women in computing research careers. The CRA-W especially encourages women to pursue Ph.D.s in computing in order to increase the proportion of women eligible for senior leadership roles. As of 2014, approximately 21% of doctoral awardees in computer science are women (National Science Foundation, 2016c). Because so few women engage in computing research career paths to begin with, it is critical to retain the small number of women in these fields. The CRA-W focuses on encouraging women who have chosen to pursue a computing career to stay the course, reach their full potential, and help increase the number of successful women role models in computing research.

Unfortunately, encouraging women to persist in computing can be an uphill battle. Prior research indicates belonging to an underrepresented or negatively stereotyped group in achievement settings, as is the case for women in computing, can lead individuals to psychologically distance themselves from that domain in order to protect the integrity of the self (Crocker and Major, 1989; Major and O'Brien, 2005; Aronson and Rogers, 2008). For instance, women graduate students in computer science may witness but not be included in camaraderie among their male peers and men faculty members. As a result, women graduate students may feel socially isolated in their program and seek camaraderie in other areas of their life (e.g., social circles in non-computing activities). As a result, women may come to place less value on the computing aspect of their lives, compared to other activities where they feel valued, thereby protecting their self-worth (Crocker and Major, 1989; Steele, 1997). Although this strategy may be self-protective, disengaging from one's computing identity may hamper women's potential for professional success. This is because when domain identification is high, positive outcomes in that domain are self-relevant and rewarding, thereby motivating persistence (Finn, 1989; Steele, 1997). Thus, women who distance their identity from computing may miss out on opportunities to feel good about their achievements in computing. This may, in turn, cause women to leave computing in pursuit of other fields that allow women to more fully develop their identity and sense of self-worth. In sum, to promote women's persistence in computing, it is important that computing is deeply integrated into their identity.

One barrier to women integrating computing into their identity is the cultural belief that computing intelligence is inborn and immutable. People who hold this belief system are called “entity theorists” (Dweck, 2006); these people are particularly likely to respond to negative feedback by becoming disheartened, losing interest in, and avoiding that particular domain (Grant and Dweck, 2003; Blackwell et al., 2007; Stout and Dasgupta, 2013). Thus, because academic settings present ample opportunity for negative feedback (e.g., critique from professors; low exam scores), students who endorse an entity theory of intelligence are particularly “at risk” of psychologically disengaging (i.e., dis-identifying) from academics (Hong et al., 1999). Because women are scarce in computing, it is important to mollify women's existing entity beliefs about computing. Importantly, research indicates entity beliefs can indeed shift so that intelligence is viewed as a muscle that can grow with time and effort (Dweck, 2006). Thus, interventions aimed at retaining women in computing should provide women with evidence that, with time and effort, success in computing is achievable for women and men alike, and women belong in computing just as much as men do. In the section that follows, we outline one intervention that aims to do just that.

## The grad cohort intervention

Grad Cohort is an intervention that has been orchestrated and run by the CRA-W annually since 2004. The number of women who have attended Grad Cohort has grown steadily over the years, ranging from 102 in 2004 to 511 in 2016. Attendees are primarily women enrolled in Ph.D. programs, with a small percentage of women enrolled in Terminal M.S. programs (e.g., 4% of participants in the 2016 cohort). At the 2-day long workshop, participants listen to presentations from and interact with 20 to 25 computing-related researchers and professionals who are women. These women role models are solicited using an internal list maintained by the CRA-W. The list includes full professors and senior level researchers who are well known for their research accomplishments; many have served as Grad Cohort speakers before and/or are CRA-W committee members. Although the content of the workshop is modified slightly from year to year, the general themes at Grad Cohort have remained consistent over the years. Specifically, through a series of presentations and panels, senior women share information on graduate school survival skills (e.g., developing a productive working relationship with one's advisor; building self-confidence), as well as more personal information and insights about professional development (e.g., strategies for navigating gender politics; balancing personal and professional goals). In addition to presentations, the workshop offers ample opportunity for questions, informal discussions, social events, and one-to-one mentoring. Through the workshop, students are able to build mentoring relationships and develop peer networks that can form the basis for ongoing interactions during their graduate careers. The workshop explicitly focuses on building community by exposing women graduate students to successful senior women in their field and enabling an opportunity to enhance their own peer networks. To the authors' knowledge, no other mentorship program of this nature exists for women graduate students in computing programs.

Although the workshop content does not explicitly focus on the factors observed in the current research, it makes sense at a theoretical level that the workshop might have an impact on all of our factors of interest. For one, the workshop intentionally exposes participants to many successful women role models in order to motivate participants to become the next generation of senior researchers. Thus, we would expect women to aspire to be successful in their own careers, and *become well known in their field*. Participants are also exposed to role models who are “giving back” to the community of women in computing. All speakers at Grad Cohort are volunteers who are dedicated to mentoring women computing students. Thus, participants may also aspire to be like those role models by wishing to *give back to their community*. Further, Grad Cohort exposes participants to an abundance of peers and role models who are women in computing, providing concrete evidence that women can be successful in computing careers. Participants likely get the sense that computing aptitude need not be biologically predetermined—women and men alike can be competent in computing. Thus, participants may be particularly *unlikely to hold entity beliefs* about the nature of computing aptitude. Together, the Grad Cohort experience is designed to give women the tools they need to overcome challenges and be successful in a computing career so that computing is a strong part of women's sense of “who they are,” or their *identity*.

## Preliminary results for grad cohort

The Computing Research Association's social science research and evaluation center, the Center for Evaluating the Research Pipeline (CERP), has assessed the immediate impact of Grad Cohort on women's commitment to computing since 2014. Prior work by CERP has found that immediately after Grad Cohort, participants report (a) greater confidence in their ability to become leaders in their field (Cundiff et al., 2014; Stout and Wright, 2015); (b) a stronger computing identity (Wright and Stout, 2016); and (c) stronger beliefs that negative feedback and setbacks are opportunities for growth (i.e., growth mindset; Cundiff et al., 2014; Stout and Wright, 2015). In addition, open ended feedback from participants, collected by the Computing Research Association, indicates women may come away from Grad Cohort feeling prepared to face hurdles head on:

“[At Grad Cohort], the session about being a woman in computing changed my life. I thought I was the only one dealing with this pressure and it is so helpful. Now I understand that differences will always be there and that my job is learning how to succeed in that environment. The session about self-confidence is another session that I will never forget; understanding that a successful career can be accompanied with failures motivates me to stand up even when I feel that I can't.” – *Grad Cohort Participant, 2012*

Grad Cohort may also encourage women to “pay it forward” by mentoring younger women:

“I am more confident to talk to other younger students and give advice about these kind of issues and make them aware that they are not alone and that we are here to support each other!” – *Grad Cohort Participant, 2015*

Thus, there is existing evidence that Grad Cohort has an immediate, positive impact on the degree to which women believe they can be successful leaders in the field, increases interest in giving back to the community, alleviates entity beliefs (i.e., promote a growth mindset), and boosts women's computing identity. But what is the shelf life of these benefits? Is there lasting impact of the workshop on women's professional goals and self-concept? And, how do women's professional goals and self-conceptions compare to those of men? If gender disparities exist, does Grad Cohort narrow these gaps? The current work was designed to address these questions.

## Overview of the current work

In the current work, our research question is *What is the long-term impact of Grad Cohort on women compared to women who have not participated in the workshop, and men?* To answer this question, we used CERP's unique infrastructure, which works with a network of computing departments across the U.S. who distribute CERP's survey instrument to graduate students. This methodology allows CERP to collect data from a large sample of graduate students, some of whom are past Grad Cohort participants, but most of whom are non-participants. To assess long-term impact of the workshop for participants vs. non-participants, we measured dedication to (a) becoming well-known in their field, and (b) using one's work to give back to the community. We also measured students' identification with computing, which refers to one's self-definition, or the degree to which one feels their computing career pursuit is an important element of “who they are.” Finally, we measured students' beliefs about the nature of computing intelligence, namely, the degree to which women viewed computing aptitude as an innate, unchangeable characteristic (i.e., *entity beliefs*). We also observed whether the typical relationship between students' entity beliefs and identification with computing existed in our sample, where stronger entity beliefs predict weaker identification with computing. We then assessed whether the Grad Cohort intervention would interrupt this relationship. That is, we thought it possible that Grad Cohort participants would show strong identification with computing, even if they held strong entity beliefs. In this way, women, who are typically a vulnerable population in computing, would be protected against the (typically pernicious) notion that computing ability is inborn.

## Method

### Grad cohort participant selection

Recruitment methods for Grad Cohort participants simulate random assignment as much as is possible outside of the laboratory. Announcements advertising Grad Cohort are disseminated to institutions affiliated with the Computing Research Association (CRA)^1^, which includes more than 200 academic institutions across the United States and Canada. Dissemination within each department is ad hoc (e.g., department chairs distribute emails to students; information is conveyed via word of mouth). Email announcements are also distributed among women's groups within the computing research field. Thus, announcements are distributed to a broad array of academic departments and special interest groups.

Women then apply to participate in Grad Cohort. The workshop is primarily funded through annual donations from academic, industry, and other computing organization sponsors; the total amount donated per year determines how many students can be accepted. Women are eligible to participate if they are a 1st, 2nd, or 3rd year graduate student, with preference given to students who have not attended before. The applicant's GPA is not considered during the application process, so priority is not given to students based on academic merit. The result is a broad sample, maximizing the numbers of women graduate students who can attend at least once during their early graduate school years, and the number of institutions that are represented regardless of size or rank.

### Research participants

During the fall of 2015, CERP distributed a survey to 74 computing departments that awarded graduate degrees. One-thousand-three-hundred-ninety-five students enrolled in a Ph.D. program in a computing field^2^ completed the survey. Of those students, 134 were women who had participated in Grad Cohort in the past^3^ and 1199 were non-participants (*n* = 293 women, *n* = 874 men, *n* = 9 non-binary gender identity, and *n* = 23 with no gender data)^4^. Because students' binary gender identity (i.e., women vs. men) was central to our research questions, we excluded individuals who identified as either non-binary or had missing gender data from our analysis.

Among participants, we opted to exclude women from this study who had only participated in the 2015 workshop (*n* = 41). For these women, it had only been approximately 6 months between their only experience with the workshop and the time we distributed the survey discussed here. Excluding this group of women from analyses allowed for the most stringent test of long-term effects of Grad Cohort on women available in our dataset. Excluding these women, as well as women who had missing data for our main variables of interest (*n* = 9), resulted in *n* = 84 past participants for analysis. Within our final sample of Grad Cohort participants, the amount of time that had elapsed since their first Grad Cohort experience ranged from 18 to 78 months [mean (*M*) = 31.57, standard deviation (*SD*) = 14.28], with 63% having participated one time, 32% having participated two times, and 5% having participated three times.

We used a propensity score matching procedure to generate two separate comparison groups for our analyses: women non-participants and men. Groups were matched on the following variables:

- Institution type: respondents reported their academic institution (all doctoral granting); we coded research activity for each institution ranging from moderate to very high using the Carnegie classification system^5^.
- Expected graduation date: respondents reported the year they expected to complete their Ph.D. program.
- Race/ethnicity: respondents selected race/ethnic categories that applied to them, resulting in the following nine categories: Arab, Middle Eastern, Persian; Asian or Asian American; Black or African American; Hispanic or Latina; Native American; Native Hawaiian or other Pacific Islander; White or Caucasian; Mixed race/ethnicity; and other.
- U.S. citizenship status: respondents indicated whether they were a U.S. citizen, permanent resident, temporary visa holder, or “other” (e.g., dual citizenship).
- Age: respondents reported their age in years.
- Terminal M.S. degree holder: respondents indicated whether or not they had completed a terminal M.S. degree prior to enrolling in their Ph.D. program.

The treatment group (i.e., Grad Cohort participants) was matched to each comparison group using 1:1 nearest neighbor matching (Rosenbaum, 2002; Austin, 2011). After matching was completed, the matched groups were compared on their similarity in terms of the matching variables using chi-squared tests and *t*-tests as applicable. These tests showed the matched samples did not significantly differ on the matching variables.

Within our full matched sample (*N* = 252; *n* = 84 participants, *n* = 84 women non-participants, *n* = 84 men non-participants), 88% was enrolled at a doctoral research university with very high research activity, 8% was enrolled at a doctoral research university with high research activity, and 4% was enrolled at a doctoral research university with moderate research activity, according to the Carnegie classification system. Eight percent of our sample expected to graduate in 2015, 33% expected to graduate in 2016, 29% expected to graduate in 2017, 18% expected to graduate in 2018, and 12% expected to graduate in 2019 or later. The racial/ethnic distribution of our sample was as follows: 5% Arab, Middle Eastern, Persian; 24% Asian or Asian American; 3% Black or African American; 5% Hispanic or Latino(a); 57% White or Caucasian; 5% Mixed race/ethnicity; and 1% other. Fifty-nine percent of the sample was U.S. citizens, 5% was non-U.S. citizens with permanent residency, 34% was non-U.S. citizens with a temporary visa, and 2% was other (e.g., dual citizen, etc.). The median age of our sample was 28. Forty-three percent of our sample had completed a Terminal M.S. before entering their current Ph.D. program, and 57% of our sample entered their current Ph.D. program without already having completed a Terminal M.S. degree.

### Procedure

Grad Cohort participants and non-participants were invited to complete an online survey via (a) an email invitation sent by their department chair or an administrative staff person in their department or (b) a direct invitation from CERP. Incentive for completing the survey was entry in a raffle to win a $100 gift card. Questions relating to students' professional goals, computing identity, and entity belief orientation were embedded within the survey. This study was reviewed and approved by an independent IRB, Solutions IRB. The IRB waived the requirement for written informed consent.

### Measures

#### Professional goals: becoming well known, and giving back to the community

Students were asked “How important to you is it that your future career allows you to do each of the following?,” using a scale of (1) *strongly disagree* to (5) *strongly agree*, and presented with a series of goals. A single item of interest was “become well-known in my field.” Also of interest was a set of goals pertaining to students' interest in giving back to their community included the following: “give back to my community”; “have social impact”; and “be a role model for people in my community.” The latter three items reliably measured a single construct (Cronbach's alpha = 0.81), so were averaged to create a composite measure.

#### Computing identity

Students were asked to rate the degree to which they agreed with the following statement, using a scale of (1) *strongly disagree* to (5) *strongly agree*: “Computing is a big part of who I am”; “I see myself as a ‘computing person’ ”; “Computing is not very important to me” (reverse scored); “I am interested in learning more about what I can do with computing”; and “Using computers to solve problems is interesting.” Items had good internal reliability (Cronbach's alpha = 0.80), and were aggregated to create a composite measure of computing identity.

#### Entity orientation

To measure entity orientation, we measured students' agreement with the following statements using a scale ranging from (1) *strongly disagree* to (5) *strongly agree*: “People have a certain amount of computing ability that really can't be changed”; “People can't really change how good they are in computing”; and “People can learn new things, but they can't change their basic ability to do computing.” These items formed a reliable index of entity orientation (Cronbach's alpha = 0.86), so we aggregated them to create a composite index.

## Results

### Professional goals

#### Becoming well known in one's field

**Figure 1:**
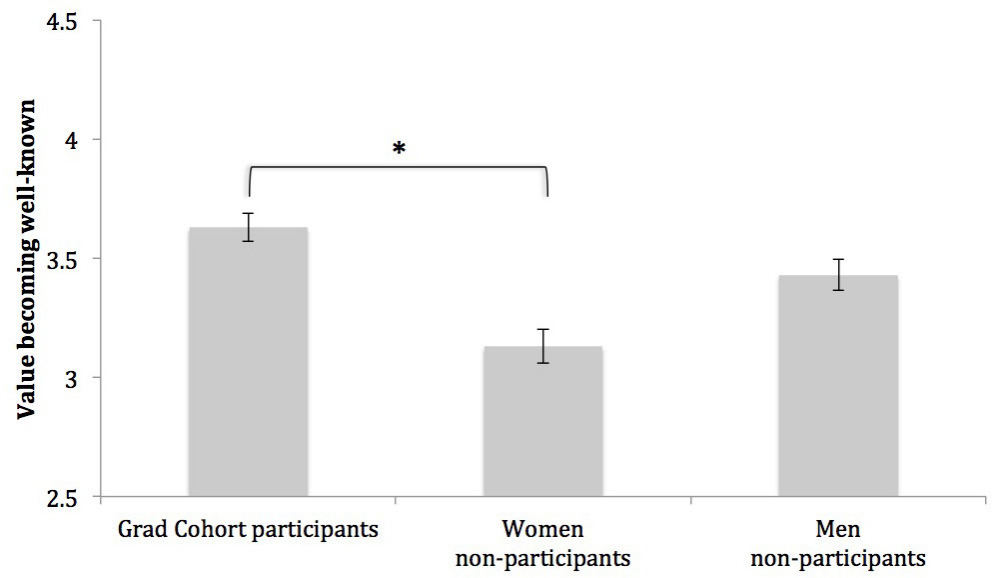
**Group differences in the degree to which students value becoming well known in their field**. Note. Bars represent group means; intervals at the top of bars represent standard errors of the mean. Scale values range from *(1) Low* to *(5) High*. ^*^Indicates group means are significantly different, *p* < 0.05.

As a group, students valued becoming well known in their field at a level just above the midpoint of our scale (3.00), *M* = 3.40, *SD* = 1.27. To assess whether students differed in their desire to become well known, we ran a one-way Analysis of Variance (ANOVA) on this measure, treating a three level student variable (Grad Cohort participants, women non-participants, men non-participants) as a between subjects factor. The degree to which students valued becoming well known in their field differed across the three groups, *F*_(1, 249)_ = 3.34, *p* < 0.05, η^2^ = 0.03. Post-hoc Dunnett tests revealed Grad Cohort participants placed more value on becoming well known in their field than women non-participants (Grad Cohort participants: *M* = 3.63, *SD* = 1.15; women non-participants: *M* = 3.13, *SD* = 1.40), *p* < 0.05, *d* = 0.39, but Grad Cohort participants and men did not differ in this value (Grad Cohort participants: *M* = 3.63, *SD* = 1.15; men non-participants: *M* = 3.43, *SD* = 1.23), *p* = 0.48, *d* = 0.17. See Figure 1 for a graph of this effect. Because men vastly outnumber women in leadership roles in computing (and outside of computing), this finding is particularly promising.

#### Giving back to the community

**Figure 2:**
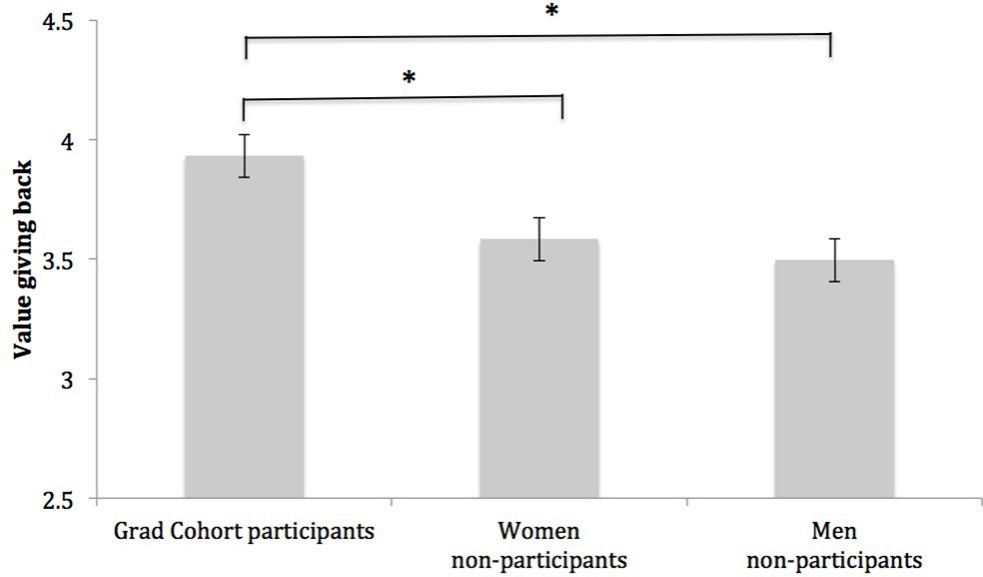
**Group differences in the degree to which students value giving back to their community**. Note. Bars represent group means; intervals at the top of bars represent standard errors of the mean. Scale values range from *(1) Low* to *(5) High*. ^*^Indicates group means are significantly different, *p* < 0.05.

Among all students in the sample, interest in giving back to the community was above the midpoint (3.00), *M* = 3.67, *SD* = 0.89. However, the degree to which students valued this goal differed across groups, *F*_(1, 249)_ = 5.89, *p* < 0.01, η^2^ = 0.05. Specifically, post-hoc Dunnett tests indicated Grad Cohort participants placed more value on giving back to the community (*M* = 3.93, *SD* = 0.84) than women non-participants (*M* = 3.58, *SD* = 0.90), *p* < 0.05, *d* = 0.40, and men non-participants (*M* = 3.50, *SD* = 0.88), *p* < 0.01, *d* = 0.50. See Figure 2 for a graph of this effect. As will be discussed in detail below, this finding suggests Grad Cohort may be fostering a desire to “pay it forward,” so that women take an active role in mentoring the next generation of computing researchers.

#### Computing identity

**Figure 3:**
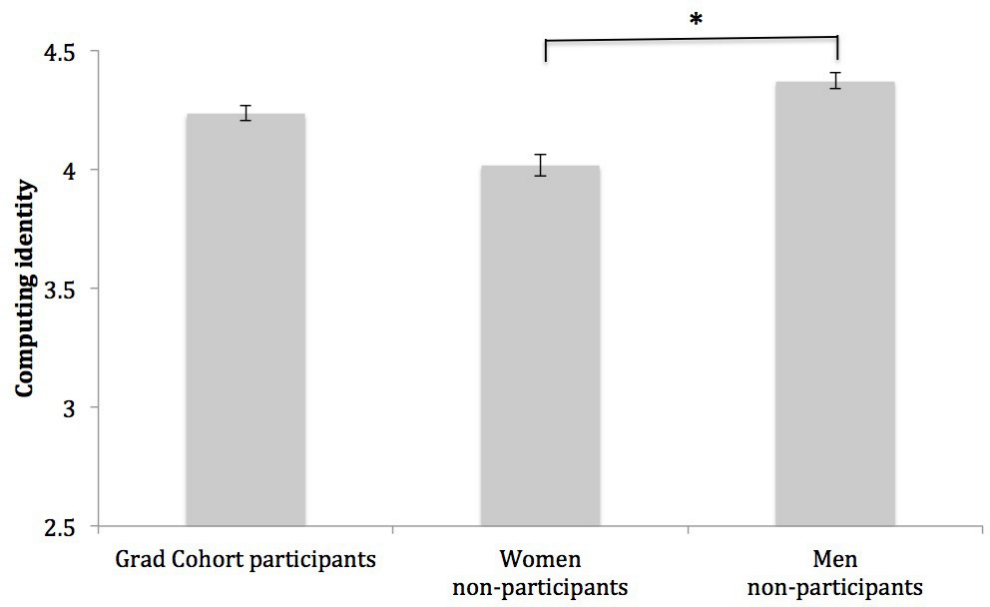
**Group differences in computing identity**. Note. Bars represent group means; intervals at the top of bars represent standard errors of the mean. Scale values range from *(1) Low* to *(5) High*. ^*^Indicates group means are significantly different, *p* < 0.05.

As a group, students identified quite strongly with computing; this was evident by the fact that students' mean identification score was well above the midpoint of the scale (3.00), *M* = 4.21, *SD* = 0.69. This is not surprising, given that students in our sample were Ph.D. students and had invested a significant amount of time and energy into studying computing during the past several years. However, computing identity differed among the three groups of students, *F*_(1, 249)_ = 5.71, *p* < 0.01, η^2^ = 0.04. Post-hoc Dunnett tests revealed whereas non-participant women identified with computing less strongly than men (women non-participants: *M* = 4.02, *SD* = 0.77; men non-participants: *M* = 4.36, *SD* = 0.72), *p* < 0.01, *d* = 0.46, Grad Cohort participants identified with computing to a statistically equivalent degree as men (Grad Cohort participants: *M* = 4.23, *SD* = 0.63; men non-participants: *M* = 4.36, *SD* = 0.72), *p* = 0.33, *d* = 0.19. These results suggest Grad Cohort may be alleviating a gender gap in students' computing identity. See Figure 3 for a graph of this effect.

### Entity orientation

**Figure 4:**
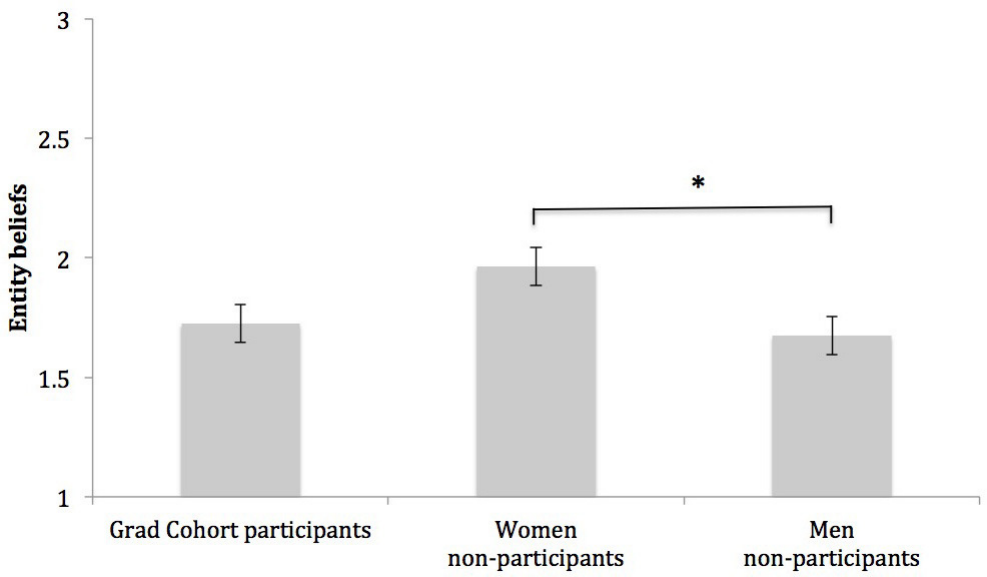
**Group differences in entity beliefs**. Note. Bars represent group means; intervals at the top of bars represent standard errors of the mean. Scale values range from *(1) Low* to *(5) High*. ^*^Indicates group means are significantly different, *p* < 0.05.

Next, we assessed students' entity belief system, and found, as a group, students held entity beliefs well below the midpoint of our measure (3.00), *M* = 1.79, *SD* = 0.78. However, the degree to which students held entity beliefs about the nature of computing did differ by student group, *F*_(1, 249)_ = 3.36, *p* < 0.05, η^2^ = 0.03. Specifically, post-hoc Dunnett tests indicated non-participant women held stronger entity beliefs than men (women non-participants: *M* = 1.96, *SD* = 0.82; men non-participants: *M* = 1.67, *SD* = 0.81), *p* < 0.05, *d* = 0.36, but Grad Cohort participants and men did not differ in their endorsement of entity beliefs (Grad Cohort participants: *M* = 1.73 *SD* = 0.67; men: *M* = 1.67, *SD* = 0.81), *p* = 0.87, *d* = 0.07. See Figure 4 for a graph of this effect.

### The relationship between entity orientation and computing identity

**Figure 5:**
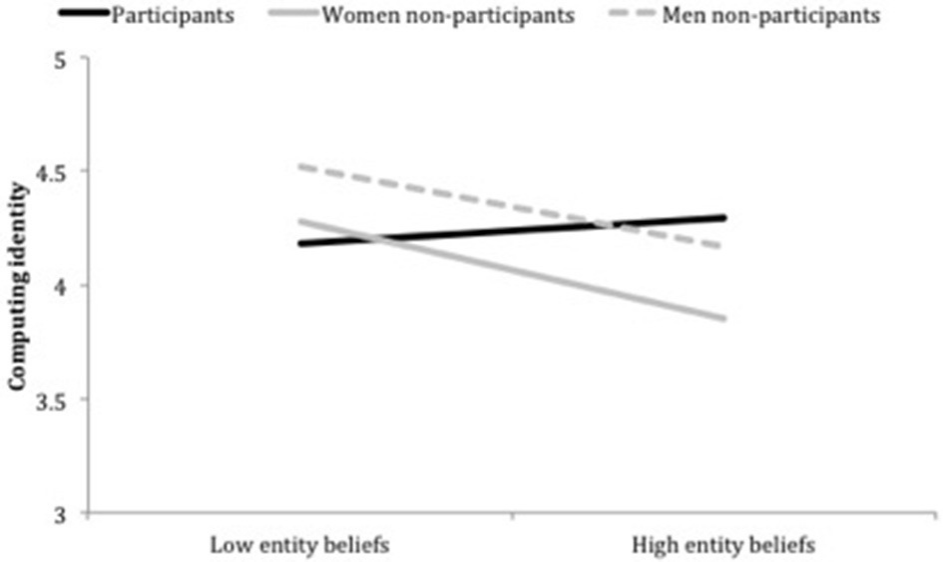
**Computing identity as a function of entity orientation and student group**. Note. Low and High entity orientation represent 1 SD below and 1 SD above the mean for entity orientation, respectively.

We next observed the relationship between entity orientation and computing identity for students, and whether this relationship differed across the three student groups. We expected to find stronger entity beliefs would predict weaker identification with computing for students in general. Indeed, entity orientation was negatively correlated with computing identity for the full sample, *r* = −0.21, *p* < 0.01. Importantly we thought it possible that Grad Cohort participants would not show a relationship between these two variables, suggesting Grad Cohort may protect students' identity from entity beliefs. To test this, we regressed computing identity (the dependent variable) on entity beliefs (mean centered), student group (two dummy-coded variables computed separately for the women non-participants and men non-participants, treating Grad Cohort participants as the reference group), and their interaction terms. The overall model was statistically significant, *R*^2^ = 0.10, *F*_(5, 243)_ = 5.65, *p* < 0.001. We found two significant Entity Belief × Student Group interactions: one comparing Grad Cohort participants to women non-participants, β = −0.24, *B* = −0.35, *SE* = 0.14, *p* < 0.05, and a second comparing Grad Cohort participants to men, β = −0.20, *B* = −0.30, *SE* = 0.14, *p* < 0.05^6^. See Figure 5 for a graph of these interaction effects.

To interpret this interaction effect, we examined whether the effect of entity orientation on computing identity differed for the three student groups using conventional dummy coding protocol where our reference group was zero and the non-reference group was 1 (Aiken and West, 1991). In doing so, we found stronger entity beliefs significantly predicted a weaker computing identity among women non-participants, β = −0.31, *B* = −0.27, *SE* = 0.09, *p* < 0.01, and men non-participants, β = −0.25, *B* = −0.23, *SE* = 0.09, *p* < 0.05. However, entity beliefs did not predict Grad Cohort participants' computing identity, β = 0.08, *B* = 0.07, *SE* = 0.11, *p* = 0.51.

We also examined the two-way interactions by observing group differences in computing identity at high and low levels of entity beliefs by re-running our original regression model using two new iterations: (1) centering entity orientation at 1 standard deviation below mean entity orientation and (2) centering entity orientation 1 standard deviation above mean entity orientation (Aiken and West, 1991). Using this strategy, we first compared Grad Cohort participants to women non-participants (solid black and solid gray lines in Figure 5), and found when entity beliefs were low (negative portion of the x axis in Figure 5), women identified with computing to a statistically equivalent degree, β = 0.07, *B* = 0.10, *SE* = 0.15, *p* = 0.54. However, when entity beliefs were high (positive portion of the x axis in Figure 5), non-participants identified with computing significantly less than Grad Cohort participants, β = −0.30, *B* = −0.44, *SE* = 0.15, *p* < 0.01. This finding suggests Grad Cohort may be protecting women's computing identity from entity beliefs; we discuss implications for this finding in the Discussion section.

Next, we compared Grad Cohort participants to men non-participants (solid black and dashed gray lines in Figure 5), and found when entity beliefs were low (negative portion of x axis in Figure 5), men identified with computing to a significantly greater degree than Grad Cohort participants, β = 0.23, *B* = 0.33, *SE* = 0.14, *p* < 0.05. But, when entity beliefs were high (positive portion of x axis in Figure 5), men and Grad Cohort participants identified with computing to a statistically equivalent degree, β = −0.09, *B* = −0.13, *SE* = 0.16, *p* = 0.43. Thus, although Grad Cohort participants identified with computing to a similar degree as men when students held strong entity beliefs, men identified with computing to a greater degree than Grad Cohort participants when students held weak entity beliefs.

## Discussion

Our research assessed the long-term outlook for women in computing who have participated in the CRA-W's Grad Cohort mentorship workshop, compared to women and men who have never participated in the workshop. First, we found past Grad Cohort participants were more interested in becoming well-known in their field than women who had not participated in Grad Cohort, and were more interested in “giving back” to their communities than their peers (both women and men). To the degree that women's “community” consists of other women—particularly young women—this finding is important as computing seeks to engage dramatically more girls and young women in the field. That is, when well-known women computing experts reach out and mentor younger women, those younger women are exposed to computing experts who “look like them.” As will be expanded upon below, seeing competent women role models can help inoculate women's self-concept from the cultural stereotype that computing is for boys. Thus, women who have participated in Grad Cohort may be particularly motivated to build a public persona, and “pay it forward” in order to help build diversity into the next generation of computing professionals.

We also found non-participant women reported computing was a less important element of “who they are” (i.e., their identity) compared to men. Of note, Grad Cohort participants' computing identity was at parity with men. This finding is consistent with existing research documenting that women identify with fields less than men when women are underrepresented and/or marginalized in those fields, but women identify with fields to an equal degree as men when both genders are represented and valued (Stout and Dasgupta, 2011; Stout et al., 2011; Dasgupta and Stout, 2014). Ph.D. students have achieved a great deal in order to gain acceptance into a Ph.D. program, and are on track to achieve a great deal more (completing a dissertation; publications; contributing to innovations in computing research). As such, it is important that students connect these achievements with their sense of self-worth in order to remain engaged in their career track. This is particularly important for women, who are strikingly underrepresented in computing careers. That is, a strong link between women's sense of self and computing can help make their achievements personally meaningful, motivating, and potentially keep women on track to become the next generation of leaders in computing research.

We also examined the degree to which students held entity beliefs about the nature of computing intelligence. This belief system is known to be maladaptive, particularly when individuals are faced with negative feedback, which is common during graduate training (Dweck, 2006). For individuals with entity beliefs, negative feedback can lead to discouragement and disengagement (Hong et al., 1999); thus, a rejected manuscript submission or criticism from one's advisor might send the message that one is intrinsically incapable of success. This chain of events is particularly undesirable when those individuals are women in computing, because women are so scarce in the field. Our findings suggest Grad Cohort may be alleviating the negative impact of entity beliefs on women's self-concept. Specifically, whereas holding strong entity beliefs about computing intelligence was related to a weak computing identity among women and men non-participants, there was no such relationship between entity beliefs and computing identity for Grad Cohort participants. We believe this finding is particularly important for retaining women in computing; moreover, this finding is reminiscent of research on other interventions for women that help inoculate women's self-concept (identity; belonging) from negative stereotypes about women's technical aptitude (see Dasgupta, 2011). That is, although negative stereotypes about women's innate technical ability continue to exist worldwide (Miller et al., 2015), and those stereotypes can lead women to underperform and psychologically disengage from those fields (Spencer et al., 1999; Stout et al., 2011; Stout and Tamer, 2016), learning environments can be modified to protect women's achievement and engagement when confronted with gender stereotypes. For instance, the presence of women teachers in calculus classes can foster a sense of enjoyment and belonging in math, even when women know negative stereotypes about women's math abilities exist (Stout et al., 2011). Other research indicates women feel strong self-efficacy and belonging in computing classrooms that foster collaboration, even when those women students are aware of stereotypes that computing is “for men” (Stout and Tamer, 2016). Because Grad Cohort shares characteristics with the studies outlined here (i.e., showcasing competent, successful women role models in computing; a sense of togetherness and support among participants), it makes sense that Grad Cohort participants' computing identity appears to be protected against beliefs that computing ability is biologically predetermined.

In summary, our results highlight the importance of same-group role models for underrepresented individuals (see Dasgupta, 2011; Stout et al., 2011). These role models can make the path to professional success for underrepresented groups more clear by (a) providing mentoring that speaks to those groups' unique experiences and (b) serving as an example of someone from that group who has succeeded (i.e., “If they can do it, I can do it too”). These role models serve as counterevidence for cultural stereotypes about natural aptitude. That is, cultural stereotypes suggest technical aptitude is innate; a related stereotype is that women are “naturally” less technically apt than men (see Miller et al., 2015). This stereotype can be debunked by showing concrete opposing evidence, such as successful, hard-working women in technical fields. Like the Grad Cohort intervention, other interventions aiming to promote success among underrepresented groups in technical fields should showcase same-group role models who symbolize the fact that hard work as opposed to inborn aptitude is the key to success in the field.

### Limitations and future directions

One limitation of the current work is its strictly quantitative focus. Taking a mixed-methods approach involving qualitative and quantitative data would strengthen the work. Future directions for Grad Cohort evaluation at CERP include directly following up with Grad Cohort participants to ask for a retrospective account of particularly impactful elements of the workshop. This inquiry will be qualitative so that we can develop hypotheses about the reasons why Grad Cohort is beneficial to women, and then test those hypotheses through our comparative survey research.

Attention to racial and ethnic diversity is notably absent in the current work. Unfortunately, our sample of Grad Cohort participants was not large enough to ask intersectional research questions [e.g., is Grad Cohort particularly impactful for a particular racial group(s)]. Recent research on women's self-efficacy immediately after Grad Cohort suggests Asian and White women may benefit more than Black, Hispanic/Latina, and Middle Eastern women (Wright and Stout, 2016). We interpret this finding with caution, given that sample sizes in this particular analysis were small for some groups (e.g., Hispanic/Latina *n* = 17). Nonetheless, intersectional analysis is a critical next step to fully understanding the impact of Grad Cohort on women's commitment to computing careers, especially given the doubly underrepresented status of women of color in computing.

We acknowledge that women who participate in Grad Cohort may be fundamentally different than women who do not participate in Grad Cohort. Although we analytically controlled for a variety of factors that might predict women's professional goals and engagement in computing (institution characteristics, respondent's expected graduation date, race/ethnicity of the respondent, U.S. citizenship status, age, and whether or not the respondents had entered their Ph.D. program with a terminal master's degree), there are other variables that we were not able to control for that could have contributed to our results. For instance, Grad Cohort participants were recruited through announcements to departments affiliated with the Computing Research Association (CRA). Although we were able to control for the size and level of research activity of participants' vs. non-participants' home institutions, we were not able to control for the climate of those institutions that may have had an impact on our results. That is, the CRA and its affiliate institutions may both value diversity initiatives to a greater degree than institutions unaffiliated with the CRA. This discrepancy may foster different climates for women, such that CRA affiliate climates are warmer and non-affiliate climates are chillier. Thus, women at departments unaffiliated with the CRA may dis-identify with computing to a greater degree, and hold stronger entity beliefs about computing aptitude than Grad Cohort participants simply because non-participants' departments lack sensitivity to gender issues in computing. Because we do not have measures of our sample's professional goals, identity, and entity beliefs prior to the Grad Cohort intervention, we cannot be sure whether this speculation is true. However, our prior research documents increases in women's leadership self-efficacy, computing identity, and growth mindset after Grad Cohort compared to immediately before the workshop (Cundiff et al., 2014; Stout and Wright, 2015; Wright and Stout, 2016). This work suggests Grad Cohort may be at least in part responsible for the benefits documented in the current research.

It is also important to note the type of women who participates in Grad Cohort may be fundamentally different from non-participants in other ways. For instance, non-participant women may actively choose not to apply to participate in Grad Cohort, either because they feel they are self-sufficient enough without the workshop or because they do not want to be associated with the workshop. Or, women may have applied for but not been accepted to Grad Cohort. Alternatively, participants may be more proactive than women who do not apply to participate in Grad Cohort. Regarding the latter possibility, we take some comfort in the fact that the CRA-W does not use GPA as a means of selecting Grad Cohort participants, as GPA can serve as a proxy for motivation and diligence. We also believe our propensity score matching technique may have controlled for participants' vs. non-participants' proactive nature to some degree. For instance, students' home institutions may also serve as a proxy for student motivation (i.e., larger schools with stronger research programs may attract more ambitious and competitive students than less research intensive schools). Further, past research on the short term benefits of Grad Cohort indicate women get a boost in their confidence to become leaders after the event (Cundiff et al., 2014; Stout and Wright, 2015). This provides some evidence that Grad Cohort may be enhancing women's commitment to becoming the next generation of leaders in computing.

The benefits of randomized assignment in order to conduct more rigorous social science research need to be weighed against the benefits of including as many women as are interested in the Grad Cohort intervention. Grad Cohort provides an opportunity for women during an already stressful and isolating period of life (i.e., graduate school) to connect with other women who share a passion for computing. The CRA-W is expressly interested in sharing the Grad Cohort experience with as many women as possible, and has reservations about intentionally excluding women from Grad Cohort to generate a control condition for research. Knowing this, we view the current research findings as promising evidence that the program may have lasting impact on women's engagement in the field of computing. Given this, the CRA-W's long standing Grad Cohort program may help fortify women's commitment to pursing computing research careers after earning their Ph.D. and move the needle in the direction of greater gender diversity in computer science.

## Author contributions

JS was the lead writer and analyst. BT and HW contributed to data collection and writing. LC, SD, and AH contributed to writing.

### Conflict of interest statement

The authors declare that the research was conducted in the absence of any commercial or financial relationships that could be construed as a potential conflict of interest.

## Footnotes

^1^The Computing Research Association is a non-profit organization whose goal is to advance computing research. The CRA-W is a subcommittee of the CRA.

^2^We define *computing field* as computer science, computer engineering or electrical and computer engineering, computing information systems or information systems, or other computing related field as reported by the respondent. Seventy-eight percent of the sample was computer science students (*n* = 196), and 22% was students from some other computing field (*n* = 56). While it would have been ideal to control for the type of subfield students were enrolled in when we analyzed the data, small sample sizes of students from other subfields of computing precluded our ability to do so.

^3^Past Grad Cohort participants in the survey were identified by asking the respondents whether they had attended CRA-W's Grad Cohort workshop in the past, and, if they had, what year(s) they attended the workshop.

^4^Ideally, the comparison group would consist of individuals who applied to Grad Cohort, but did not participate. However, applicant contact information was not available at the time of data collection for this project. Further, such a comparison group does not exist for men, given that men are not eligible to participate in Grad Cohort.

^5^See http://webtest.iu.edu/~carclass/demo2/methodology/basic.php for further information on how the institutions were coded using the Carnegie classification system.

^6^A second model treating women non-participants as the reference group indicated there was no significant interaction when comparing women non-participants to men non-participants, *p* = 0.71.

## References

[B1] Aiken L. S. West S. G. (1991). Multiple Regression: Testing and Interpreting Interactions. Thousand Oaks, CA: Sage.

[B2] Aronson J. Rogers L. (2008). Overcoming stereotype threat, in Positive Psychology: Exploring the Best in People, Vol. 3, ed Lopez S. J. (Westport, CT: Praeger), 109–121.

[B3] Austin P. C. (2011). An introduction to propensity score methods for reducing the effects ofconfounding in observational studies. Multiv. Behav. Res. 46, 399–424. 10.1080/00273171.2011.568786 21818162 PMC3144483

[B4] Blackwell L. S. Trzniewski K. H. Dweck C. S. (2007). Implicit theories of intelligence predict. achievement across an adolescent transition: a longitudinal study and an intervention. Child Dev. 78, 246–263. 10.1111/j.1467-8624.2007.00995.x 17328703

[B5] Camp T. (2012). Computing, we have a problem. ACM Inroads 3, 34–40. 10.1145/2381083.2381097

[B6] Cejka M. A. Eagly A. H. (1999). Gender-stereotypic images of occupations. correspond to the sex segregation of employment. Pers. Soc. Psychol. Bull. 25, 413–423. 10.1177/0146167299025004002

[B7] Cheryan S. Plaut V. C. Handron C. Hudson L. (2013). The stereotypical computer scientist: gendered media representations as a barrier to inclusion for women. Sex Roles 69, 58–71. 10.1007/s11199-013-0296-x

[B8] Crocker J. Major B. (1989). Social stigma and self-esteem: the self-protective properties of stigma. Psychol. Rev. 96, 608–630. 10.1037/0033-295X.96.4.608

[B9] Cundiff J. L. Stout J. G. Wright H. M. (2014). CRA-W Grad Cohort:Pretest/Posttest Evaluation Report. Washington, DC: Computing Research Association:

[B10] Dasgupta N. (2011). Ingroup experts and peers as social vaccines who inoculate the self-concept: the stereotype inoculation model. Psychol. Inq. 22, 231–246. 10.1080/1047840X.2011.607313

[B11] Dasgupta N. Stout J. G. (2014). Girls and women in science, technology, engineering and mathematics: STEMing the tide and broadening participation in STEM careers. Policy Insights Behav. Brain Sci. 1, 21–29. 10.1177/2372732214549471

[B12] Dweck C. S. (2006). Mindset. New York, NY: Random House.

[B13] Finn J. D. (1989). Withdrawing from school. Rev. Educ. Res. 59, 117–142. 10.3102/00346543059002117

[B14] Forbes T. (2016). The 10 Best-Paying STEM Jobs for Recent Grads. Available online at: http://www.forbes.com/pictures/efkk45ekgkh/the-10-best-paying-stem-jobs-for-recent-grads/jobs-for-recent-grads/

[B15] Grant H. Dweck C. S. (2003). Clarifying achievement goals and their impact. J. Pers. Soc. Psychol. 85, 541–553. 10.1037/0022-3514.85.3.541 14498789

[B16] Hoever I. J. van Knippeberg D. van Ginkel W. P. Barkema H. G. (2012). Fostering team creativity: perspective taking as key to unlocking diversity's potential. J. Appl. Psychol. 97, 982–996. 10.1037/a0029159 22774764

[B17] Hong Y. Y. Chiu C. Dweck C. S. Wan W. (1999). Implicit theories, attributions, and coping: a meaning system approach. J. Pers. Soc. Psychol. 77, 588–599.

[B18] Leung K. Maddux W. Galinsky A. D. Chiu C.-Y. (2008). Multicultural experience enhances creativity: the when and how. Am. Psychol. 63, 169–181. 10.1037/0003-066X.63.3.169 18377107

[B19] Major B. O'Brien L. T. (2005). The social psychology of stigma. Annu. Rev. Psychol. 56, 393–421. 10.1146/annurev.psych.56.091103.070137 15709941

[B20] Margolis J. Fisher A. (2002). Unlocking the Clubhouse: Women in Computing. Cambridge, MA: The MIT Press.

[B21] Miller D. I. Eagly A. H. Linn M. C. (2015). Women's representation in science predicts national gender-science stereotypes: evidence from 66 nations. J. Educ. Psychol. 107, 631–644. 10.1037/edu0000005

[B22] Rosenbaum P. R. (2002). Constructing matched sets and strata, in Observational Studies, ed Rosenbaum P. R. (New York, NY: Springer), 295–331.

[B23] National Science Foundation (2015a). TABLE 9-5. Employed Scientists and engineers, by Occupation, Highest Degree Level, and Sex: 2013. [NCSES Data Tables: Women, Minorities, and Persons with Disabilities in Science and Engineering]. Availaible online at: http://www.nsf.gov/statistics/2015/nsf15311/tables/pdf/tab9-5.pdf

[B24] National Science Foundation (2015b). NCSES Data Tables: Women, Minorities, and Persons with Disabilities in Science and Engineering. Available online at: http://www.nsf.gov/statistics/2015/nsf15311/tables.cfm

[B25] National Science Foundation (2016a). TABLE 5-1. Bachelor's Degrees Awarded, by Sex and Field: 2004–14. [NCSES Data Tables: Women, Minorities, and Persons with Disabilities in Science and Engineering]. Retrieved from http://www.nsf.gov/statistics/2015/nsf15311/tables/pdf/tab5-1-updated-2016-08.pdf

[B26] National Science Foundation (2016b). TABLE 6-2. Master's Degrees Awarded to Women, by Field: 2004–14. [NCSES Data Tables: Women, Minorities, and Persons with Disabilities in Science and Engineering]. Available online at: http://www.nsf.gov/statistics/2015/nsf15311/tables/pdf/tab6-2-updated-2016-08.pdf

[B27] National Science Foundation (2016c). TABLE 7-2. Doctoral Degrees Awarded to Women, by Field: 2004–14. [NCSES Data Tables: Women, Minorities, and Persons with Disabilities in Science and Engineering]. Available anline at: http://www.nsf.gov/statistics/2015/nsf15311/tables/pdf/tab7-2-updated-2016-08.pdf

[B28] National Science Foundation (2016d). TABLE 8-1. SandE Postdoctoral Fellows in Academic Institutions, by Field, Citizenship, and Sex: 2014. [NCSES Data Tables: Women, Minorities, and Persons with Disabilities in Science And Engineering]. Available online at: http://www.nsf.gov/statistics/2015/nsf15311/tables/pdf/tab8-1-updated-2016-06.pdf

[B29] Sheffield S. L. (ed.). (2004). Professionalizing women scientists, in Women and Science: Social Impact and Interaction (New Brunswick, NJ: Rutgers University Press), 127–156.

[B30] Smith S. L. Choueiti M. Pieper K. (2014). Gender Bias without Borders. Report prepared for the Geena Davis Institute on Gender in Media. Available online at: http://seejane.org/research-informs-empowers/

[B31] Spencer S. J. Steele C. M. Quinn D. M. (1999). Stereotype threat and women's math performance. J. Exp. Soc. Psychol. 35, 4–28. 10.1006/jesp.1998.1373

[B32] Steele C. M. (1997). A threat in the air: how stereotypes shape intellectual identity and performance. Am. Psychol. 52, 613–629 10.1037/0003-066X.52.6.613 9174398

[B33] Stout J. G. Dasgupta N. (2011). When he doesn't mean you: gender-exclusive language as a form of subtle ostracism. Pers. Soc. Psychol. Bull. 37, 757–769. 10.1177/0146167211406434 21558556

[B34] Stout J. G. Dasgupta N. (2013). Mastering one's destiny mastery goals promote challenge and success despite social identity threat. Pers. Soc. Psychol. Bull. 39, 748–762. 10.1177/0146167213481067 23478676

[B35] Stout J. G. Dasgupta N. Hunsinger M. McManus M. (2011). STEMing the tide: using ingroup experts to inoculate women's self-concept in science, technology, engineering, and mathematics (STEM). J. Pers. Soc. Psychol. 100, 255–270. 10.1037/a0021385 21142376

[B36] Stout J. G. Tamer B. (2016). Collaborative learning eliminates the negative impact of gender stereotypes on women's self-concept, in Proceedings for the Annual Meeting of the American Society for Engineering Education (New Orleans, LA).

[B37] Stout J. G. Wright H. M. (2015). Understanding the Immediate Impact of the CRA-W's Grad Cohort Program on Diverse Women. Washington, DC: Computing Research Association.

[B38] U.S. News World Report (2016). Best Jobs 2015. Available online at: http://money.usnews.com/careers/best-jobs/rankings

[B39] Woolley A. W. Chabris C. F. Pentland A. Hashmi N. Malone T. M. (2010). Evidence for a collective intelligence factor in the performance of human groups. Science 330, 686–688. 10.1126/science.1193147 20929725

[B40] Wright H. M. Stout J. G. (2016). The CRA Committee on the Status of Women in Computing Research (CRA-W) Grad Cohort 2016: Evaluation Report. Washington, DC: Computing Research Association.

